# The data of nanoindentation on the graphene/nickel system

**DOI:** 10.1016/j.dib.2018.01.104

**Published:** 2018-02-07

**Authors:** Yuping Yan, Shangru Zhou, Sheng Liu

**Affiliations:** School of Power and Mechanical Engineering, Wuhan University, Wuhan 430072, China

## Abstract

This article contains data related to the research article entitled “Atomistic simulation on nanomechanical response of indented graphene/nickel system” (Yan et al., 2017). There are five sets of data obtained by molecular dynamics simulations for nanoindentation of five different graphene/nickel systems, which are single nickel system, monolayer graphene on nickel system, double-layer graphene on nickel system, three-layer graphene on nickel system and four-layer graphene on nickel system. The calculated load-displacement of the five different indented systems is also listed.

**Specifications table**

**Table:** 

Subject area	Physics
More specific subject area	Two-dimensional material
Type of data	Csv file, Figure
How data was acquired	Molecular dynamics simulations
Data format	Raw
Experimental factors	None
Experimental features	None
Data source location	Wuhan, China
Data accessibility	The data is with this article

**Value of the data**

• Data presented here provide the nanoindentation mechanical properties of nickel substrate with or without graphene coverage on.
• We show the nanoindentation mechanical properties of different number of graphene layers suspended on nickel system.
• The enhancement effect of graphene on the contact stiffness and load bearing capacity of graphene/nickel system can be calculated from the data presented here.

## 1 Data

**Fig. 1:**
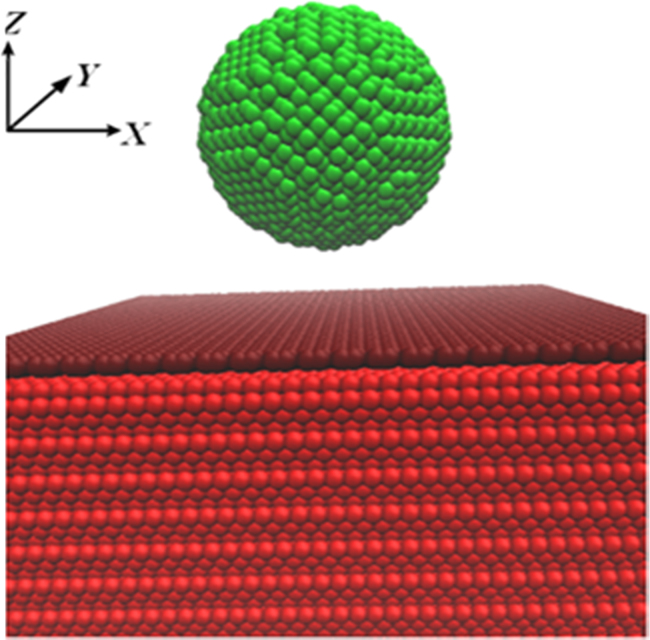
The coordinates of the model and the atomic model of indented graphene/nickel system, green for diamond spherical indenter, purplish red for suspended graphene and red for nickel substrate. (For interpretation of the references to color in this figure legend, the reader is referred to the web version of this article.)

The data presented in this article show the displacement and related load of the five different indented graphene/nickel systems (Table 1), and the load-displacement curves of graphene/nickel systems (Fig. 1).

## 2 Experimental design, materials and methods

### 2.1 Models

In our simulations, models with a graphene sheet suspended on a face-centered cubic (fcc) Ni (111) surface and diamond spherical indenters were established, as shown in Fig. 1. The structure of spherical indenter is diamond. The spherical diamond indenters radius 2.0 nm. The nickel block is cubic cell with the side length of around 5 times the indenter radius, with a well-defined graphene supported on. The spherical diamond indenters were set to be rigid. The rigid indenters were located 1.0 nm on the top of the graphene center. The number of graphene layers varies from 0 to 4. The separation between the graphene layers is 3.5 Å. The bottom two layers of the nickel substrates are set as boundary atoms, which are unaffected during the simulation and remain fixed in their initial position, serving to reduce the boundary effects.

### 2.2 Potential function

The accuracy of a potential function determines the reliability of Molecular dynamics (MD) simulation. Up to now, there are many types of force fields available, which have been parameterized to describe a variety of systems. The embedded-atoms method (EAM) potential developed by Mishin et al. [1] was used to describe the interaction between Ni atoms. Reactive empirical bond order potential (AIREBO) [2] was used to describe the intralayer carbon- carbon interaction of graphene, which gave a carbon-carbon bond length to be 0.142 nm, and agreed well with the experimental result [3]. Here, we adopted the classical Lennard-Jones (LJ) potential to describe carbon-nickel interaction between carbon atoms of diamond indenters and graphene and nickel substrate, with 0.023049 eV and 2.852 Å [4]. The van der Waals interaction between the graphene layers and indenter-graphene were modeled by the LJ interaction with 0.00284 eV and 3.4 Å [5].

### 2.3 MD setup

An energy minimization process was carried out to avoid overlaps in the positions of the atoms after models completed. The periodic boundary conditions were employed in the transverse directions (XY directions). A Nose/Hoover thermostat was applied to maintain the temperature of 300 k. The equations of motion were integrated using the Verlet algorithm [6] with a time step of 10–15 s. The system was stabilized for 100 ps to make the system reach equilibrium. The indenters were moved at a constant speed along the z-direction until to the preset indentation depth. The indent speeds varied from 25 m/s. The forces acting on the indenter were obtained by summing the forces contributed by the graphene and substrates. The data is used to analysis the effect of graphene coverage and different number of graphene layers to the nanoindentation mechanical of nickel substrate.

## Footnotes

Appendix A Supplementary data associated with this article can be found in the online version at 10.1016/j.dib.2018.01.104.
